# Functional Epistatic Interaction between rs6046G>A in *F7* and rs5355C>T in *SELE* Modifies Systolic Blood Pressure Levels

**DOI:** 10.1371/journal.pone.0040777

**Published:** 2012-07-18

**Authors:** Said El Shamieh, Ndeye Coumba Ndiaye, Maria G. Stathopoulou, Helena A. Murray, Christine Masson, John V. Lamont, Peter Fitzgerald, Athanase Benetos, Sophie Visvikis-Siest

**Affiliations:** 1 Université de Lorraine, “Génétique Cardio-vasculaire”, EA-4373, Nancy, France; 2 Randox Laboratories Ltd, Crumlin, Antrim, United Kingdom; 3 INSERM U961, Université de Lorraine, Nancy, France; 4 CHU Nancy, Brabois, Service de Gériatrie, Nancy, France; Kunming Institute of Zoology, Chinese Academy of Sciences, China

## Abstract

**Background:**

Although numerous genetic studies have been performed, only 0.9% of blood pressure phenotypic variance has been elucidated. This phenomenon could be partially due to epistatic interactions. Our aim was to identify epistatic interaction(s) associated with blood pressure levels in a pre-planned two-phase approach.

**Methods and Results:**

In a discovery cohort composed of 3,600 French individuals, we found rs6046A allele in *F7* associated with decreased blood pressure levels (P≤3.7×10^−3^) and rs5355T allele in *SELE* associated with decreased diastolic blood pressure levels (P = 5×10^−3^). Both variants interacted in order to influence blood pressure levels (P≤0.048). This interaction was replicated with systolic blood pressure in 4,620 additional European individuals (P = 0.03). Similarly, in this replication cohort, rs6046A was associated with decreased blood pressure levels (P≤8.5×10^−4^). Furthermore, in peripheral blood mononuclear cells of a subsample of 90 supposed healthy individuals, we found rs6046A positively associated with *NAMPT* mRNA levels (P≤9.1×10^−5^), suggesting an eventual involvement of *NAMPT* expression in blood pressure regulation. Confirming this hypothesis, further transcriptomic analyses showed that increased *NAMPT* mRNA levels were positively correlated with *ICAM1*, *SELL*, *FPR1*, *DEFA1-3*, and *LL-37* genes expression (P≤5×10^−3^). The last two mRNA levels were positively associated with systolic blood pressure levels (P≤0.01) and explained 4% of its phenotypic variation.

**Conclusion:**

These findings reveal the importance of epistatic interactions in blood pressure genetics and give new insights for the role of inflammation in its complex regulation.

## Introduction

Blood pressure (BP) is a heritable trait with estimates indicating that 30–70% of its variance is attributed to genetics [1], [2]. In family studies its heritability varies, according to measurement processes, from ≈31% [single-measure of systolic blood pressure (SBP) and diastolic blood pressure (DBP)], to ≈57% (long-term average of SBP and DBP phenotypes) and to ≈68% (24-hour profile of SBP and DBP) [3]. Both BP and essential hypertension (HTN) are considered polygenic traits [4]. Inflammation, blood coagulation cascade, cellular adhesion molecules and lipid metabolism appear to have significant roles [5].

The largest Genome-wide association study (GWAS) on BP including ≈200,000 individuals [6] reported 29 loci to be associated with SBP, DBP and/or essential HTN [6]. However, their genetic risk score explained only 0.9% of BP phenotypic variation [6], this representing the so-called ‘dark matter’ of genetic risk [7]. Despite the very large sample-size studies used for gene discovery, many common variants with small effects on BP may remain unidentified [8]. A large ‘hidden heritability’ of unknown nature may be explained by rare variants, structural large variants, epistatic [gene*gene (G*G)] and gene*environment (G*E) interactions [7]. We pointed out that epistatic interactions might also play an important role in discovering new genes [7]. This statement has been extensively reviewed in the last years and multi-locus methods have been developed to detect such interactions [7].

Epistatic interactions have been documented for susceptibility to cancer [9], morphology [10] and autoimmune conditions [11]. However, to date they have not been extensively studied in BP regulation. We hypothesize that the research of epistatic interactions among candidate single nucleotide polymorphisms (SNPs) represents a challenge in the investigation of disease-risk variants, as their application to high-dimensional genome-wide data exhaustively including all SNPs combinations is not yet feasible [7]. In previous candidate gene studies, we showed interesting results concerning the identification of BP candidate SNPs [12]–[16]. However, these studies were conducted in limited-sized populations.

Therefore, in the present study, we investigated BP epistasis mechanisms in a pre-planned two-phase approach gathering 8,220 European individuals. The effect of 10 candidate SNPs and then, G*G interactions between significant SNPs were assessed in a discovery population of 3,600 individuals. Highlighted epistases were replicated in 4,620 additional European individuals. We further searched for association(s) with 10 inflammation-related genes in peripheral blood mononuclear cells (PBMCs) (*LL37*, *DEFA1-3*, *FPR1, ICAM1*, *SELL*, *SELP*, *NAMPT* (*visfatin*), *LEP, TNF* and *IL-6*) [17] of a subsample of 90 supposed healthy individuals. Finally, we sought to propose a possible molecular mechanism of action.

## Materials and Methods

### Ethics Statement

All participants involved in the present study were recruited in accordance with the latest version of the Declaration of Helsinki for Ethical Principles for Medical Research Involving Human Subjects and gave written informed consent. Genetic studies protocols were approved by the local ethics committees for the protection of subjects for biomedical research: 1) the *Comité Consultatif de Protection des Personnes dans la Recherche Biomédicale de Lorraine*, Nancy, France, for populations recruited in the Center of Preventive Medicine. 2) the *Comité d’Ethique du Centre Hospitalier Universitaire de Cochin*, Paris, France, for ERA population. 3) The ethic committee of Belfast, Ireland, for population recruited in Ireland.

### Study Populations

#### Discovery population

A sample of 2,971 unrelated individuals was recruited during free medical check-ups at the Center of Preventive Medicine of Vandœuvre-lès-Nancy in the East of France. They were Caucasians, born in France for three generations and their clinical and biological data were collected at baseline before any eventual drug prescription following consultation. They were selected on the basis of the following criteria: (1) no antihypertensive drug therapy at recruitment; (2) complete clinical and genotypic data available; (3) and BP levels ranging from normotensive to stage 2 HTN (for hypertensive individuals, data were gathered before the prescription of any medication).

As our purpose was to assess BP as a continuous trait, and in order to have a proper inter-individual variability, we included individuals from the ERA cohort (*Evolution de la Rigidité Artérielle*) in the discovery population. ERA participants were selected from a Parisian cohort that had a health check-up at the *‘Investigations Préventives et Cliniques’* center. The details of this study have been previously presented [18]. Six hundred and twenty nine individuals randomly selected in ERA were incorporated in the discovery population. As no significant differences between minor allele frequencies (MAFs) of the investigated genetic variants and BP levels in these samples were found, we regrouped both discovery samples in order to perform our statistical analyses.

The corresponding samples were part of a human sample storage platform: the Biological Resources Bank ‘Interactions Gène-Environnement en Physiopathologie CardioVacsulaire’ (BRC IGE-PCV) in Nancy, East of France.

#### Replication population

We used a non-overlapping sample extracted from the BRC IGE-PCV. Altogether, 4,620 individuals with (1) no antihypertensive drug therapy at recruitment; (2) complete clinical and genotypic data for rs5355C>T in *SELE* and rs6046G>A in *F7* were available; (3) BP levels ranging from normotensive to stage 2 HTN; and (4) only European origins were analyzed (Ireland, French). Stage 3 HTN patients were also excluded in the replication population as they were treated with antihypertensive medication.

### Clinical and Biological Data Collection

SBP and DBP were measured under constant temperature (19°C–21°C) and standardized conditions (supine position) using a manual sphygmomanometer (Colonne à mercure, Mercurius) by expert nurses [18]. The recorded values were the means of 3 readings with 20 min intervals. An adjustable BP cuff was used to correct errors due to variations in arm circumference [19]. HTN was defined as SBP≥140 mmHg or DBP≥90 mmHg as recommended in the Seventh Report of the Joint National Committee on the prevention, detection, evaluation, and treatment of high BP [20]. All individuals underwent complete medical examination including anthropometric and biochemical measurements collected with standardized methods as described elsewhere [17].

### Genotyping Assays

We selected rs1799752Ins>del in *ACE*, *rs5882A>G in CETP*, rs1801133C>T in *MTHFR* rs662A>G in *PON1* and rs1800629G>A in *TNF* from the “Cardio-Vascular Disease 35” assay, a multilocus genotyping assay developed in collaboration with Roche Molecular Systems [12]. These genetic variants were candidate markers for cardiovascular disease (CVD) risk factors, specifically involved in the predisposition to essential HTN (rs1799752Ins>del in *ACE*), in the development of atherosclerotic plaques and in the progression of atherosclerosis (*rs5882A>G in CETP*, rs1801133C>T in *MTHFR* rs662A>G in *PON1* and rs1800629G>A in *TNF*) [12]. In addition, rs5355C>T in *SELE* [13], [21], rs1800790G>A in *FGB* [14], rs6046G>A in *F7* [15], rs328C>G in *LPL* [16], [22] were chose based on our previous published studies that found these SNPs associated with BP levels and/or HTN in European populations [12]–[16], [21], [22]. Finally, rs3025058T>Ins in *MMP3* was selected from an internal investigation showing a link between this genetic variant and BP levels.

A summary of investigated genetic variants (nearby gene, location, type and mutation) was shown in Supplementary Data S1.

Genomic DNA was extracted from peripheral blood samples using the salting out method [23]. Genotyping was performed using two methods in the discovery population. 1) A multilocus assay with an immobilized probe approach designed by Roche Molecular Systems, Pleasanton, California, USA [24]. After PCR amplification using pooled biotinylated primers and hybridization to sequence-specific oligonucleotide probes, two independent observers using proprietary Roche Molecular Systems image processing software performed genotype assignments. Among 2,971 individuals, discordant results (<3% of all scoring) were resolved by a third observer and if necessary, by a joint reading. 2) Evidence Investigator™ biochip designed by Randox Laboratories, Antrim, UK was used to genotype ERA participants. This genotyping assay is based on a combination of probe hybridization, ligation, PCR amplification and microarray hybridization. This unique design permits high assay multiplexing and ready discrimination between genotypes. For the validation of genotyping results, blinded replication analysis was performed on 50 common samples. Both genotyping methods gave matched results at 99% (data available on demand).

#### Replication population

Only rs5355C>T in *SELE* and rs6046G>A in *F7* were genotyped. Among all individuals; 2,059 were genotyped by Kbioscience company using the competitive allele specific PCR (KASP) chemistry coupled with a FRET-based genotyping system (http://www.kbioscience.co.uk/reagents/KASP/KASP.html). The remaining 2,561 individuals were genotyped by Roche multilocus assay as described previously.

### PolyPhen Analysis of Nonsynonymous SNPs

The prediction of nonsynonymous SNPs possible impacts on their protein structures was performed using PolyPhen [25].

### Peripheral Blood Mononuclear Cells Collection

Freshly drawn peripheral venous blood (10 ml) was collected into tubes containing EDTA (Vacutainer, Becton Dickinson) under fasting conditions. PBMCs were isolated by centrifuging on a density gradient of Ficoll as described previously and stored at -80°C until RNA extraction [26]. PBMCs bank with high recovery of lymphocytes (97.5%) was constituted as described elsewhere [26].

### RNA Extraction and qRT-PCR Analysis

Using a microarray analysis [5]; we selected the top 10 inflammation-related genes (from a total of 182 genes) having a higher expression in PBMCs of hypertensive individuals when compared with normotensives. Total RNA was isolated from PBMCs by an automated isolation procedure (MagNa Pure LC instrument). mRNA quality and stability were carefully tested [26] and reverse transcribed as previously described [26]. Quantitative real-time PCR (qRT-PCR) was performed using LightCycler instrument (Roche Diagnostics, Mannheim, Germany) with Master Plus SYBR Green I kit for all gene transcripts. *SELE* and *F7* were not quantified, as they were not expressed. Specific primers were designed using Primer Premier 3.0 software (Supplementary Data S1). All experiments were carried out in duplicates in a total reaction volume of 20 µl containing 0.5 mM of each specific primer. Negative and internal controls were included. All mRNA levels were normalized to the mRNA levels of *POL2RA*. The specificity of all PCR products was further verified by electrophoresis on 10% polyacrylamide gel (data available on demand). The clinical characteristics of the studied subsample were presented in Supplementary Data S1.

### Statistical Analyses

Statistical analyses were performed using the SPSS® statistical software version 19.0 (SPSS, Inc, Chicago, Illinois). Polymorphisms with MAF deviating from Hardy-Weinberg equilibrium (HWE) were excluded from individual analyses. In order to determine the effect of the 10 selected genetic variants on SBP and DBP assuming additive models using the common wild type as the reference group; age, gender and body mass index (BMI)-adjusted linear regressions were performed for individual association analyses. Due to multiple testing, the significance level was set at P≤5×10^−3^ in the discovery and replication populations.

#### G*G interactions

Two-locus additive epistasis was defined as significant statistical interaction between two SNPs [27] and was determined when significant interaction existed on a linear additive model adjusted for age, gender and BMI. Epistatic interactions were only tested between individually significant associated SNPs. In both populations, Bonferroni correction for multiple testing was applied. The significance level was set at P≤0.05.

#### SNP-mRNA association analysis

Linear regressions were performed to assess the effect of SNPs previously associated to SBP and/or DBP in the first stage of our analyses on mRNA levels. In epistatic conditions, an interaction term was introduced in the model. The significance level was set at P≤5×10^−3^ due to multiple testing.

#### Pearson’s correlation analyses

Pearson’s correlation was used to test the correlation between all genes expression and *NAMPT* levels (values log-transformed). The significance level was set at P≤5×10^−3^ due to multiple testing.

#### Linear regression analysis between genes expression and BP levels

Linear regression models were used to further assess the association of *SELL*, *FPR1*, *ICAM1*, *DEFA1-3* and *LL-37* with mRNA levels with SBP and DBP after adjustment for age and gender. The significance level was set at P≤0.01 due to multiple testing.

### URLs

Primer Premier 3.0 is available at: http://frodo.wi.mit.edu/primer3/.

Polyphen is available at: http://genetics.bwh.harvard.edu/pph2/.

## Results

Table 1 presents the clinical characteristics of the studied populations. According to the Seventh Report of the Joint National Committee [28], 21.8% of participants had normal BP, 32% were pre-hypertensive and 46.2% had HTN stage 1 and 2 in the discovery population (Table 1). In the replication set, 34% had normal BP, 39.8% were pre-hypertensive and 26.2% were stage 1 and 2 hypertensive (Table 1). A higher frequency of HTN was observed in the discovery compared to the replication population (46.2% vs. 26.2% respectively), which is partly due to the presence of older individuals in the discovery set.

**Table 1:** Characteristics of studied individuals.

	Discoverypopulation	Replicationpopulation
**N (% women)**	3,600 (47.4)	4,620 (43.3)
**Age (years)**	47.3±10.5	38.2±16.6
**BMI (kg/m^2^)**	25.4±3.8	24.3±4.4
**SBP (mmHg)**	136.9±20.2	130.6±20.1
**DBP (mmHg)**	84.1±13.8	77.1±16
**BP category (%)**		
**<120/80 mmHg**	21.8	34
**120–139/80–89 mmHg**	32	39.8
**≥140 and/or 90 mmHg**	46.2	26.2
**MAF (%)**		
**rs5355C>T**	16	9
**rs6046G>A**	25	25

BMI: body mass index, BP: blood pressure, SBP: systolic blood pressure, DBP: diastolic blood pressure, MAF: minor allele frequency.

Table 2 shows genetic variants associated with BP traits. We found two SNPs, rs5355C>T in *SELE* and rs6046G>A in *F7* showing associations with SBP and/or DBP respectively in the discovery population (P_discovery_≤5×10^−3^, Table 2). rs5355T allele in *SELE* was associated with decreased DBP levels (P = 5×10^−3^, β = −0.04, Table 2), whereas rs6046A allele in *F7* was associated with decreased SBP and DBP levels respectively (P = 3.7×10^−3^ and P = 8.2×10^−4^ respectively, Table 2). Both SNPs are nonsynonymous, introducing amino acid substitutions (Leu575Phe and Arg353Gln respectively). According to Polyphen, they were predicted to have a null effect on their corresponding protein structures. full individual association results with BP in the discovery and the replication population were shown in Supplementary Data S1.

**Table 2:** Genetic variants associated with blood pressure.

Chr	Gene	SNP ID	Discovery population		Replication population		P_meta_	BP trait
			P_discovery_	Beta* (mmHg)	P_replication_	Beta* (mmHg)		
1q22-q25	*SELE*	rs5355C>T	5×10^−3^	−0.04	0.86	–	0.09	DBP
13q34	*F7*	rs6046G>A	3.7×10^−3^	−0.06	8.45×10^−4^	−0.03	2.03×10^−4^	SBP
			8.2×10^−4^	−0.08	2.58×10^−7^	−0.03	9.16×10^−4^	DBP

* : Log10 transformed values.

Beta coefficients are shown for significant associations.

Chr: chromosome, SNP: single nucleotide polymorphism, MAF: minor allele frequency, Beta: coefficient in the linear regression model, BP: blood pressure, P_meta_: P meta-analysis, SBP: systolic blood pressure, DBP: diastolic blood pressure.

In order to examine whether rs5355C>T in *SELE* and rs6046G>A in *F7* may also indirectly influence BP levels, we tested their G*G interaction (Table 3). Both SNPs interacted in order to influence SBP and DBP in the discovery population (P = 0.048 and P = 0.047 respectively, Table 3A). Table 3A shows BP variations according to rs5355T allele in *SELE* and rs6046G/A genotypes in *F7* when compared to rs5355C allele in *SELE.* We found that individuals carrying rs5355T allele in *SELE* and rs6046GG in *F7* had 6.5 mmHg and 8 mmHg decrease in SBP and DBP respectively when compared with carriers of rs5355C allele in *SELE* and rs6046GG genotype in *F7* (Table 3A). In contrast, individuals carrying rs5355T allele in *SELE* and one minor allele of rs6046G>A (rs6046GA) had 6.1 mmHg and 1.2 mmHg increase in SBP and DBP respectively when compared with carriers of rs5355C allele in *SELE*, rs6046GA genotype in *F7* (Table 3A). Furthermore, carriers of rs5355T allele in *SELE* and two minor alleles of rs6046G>A (rs6046AA) had higher BP levels when compared with those carrying rs5355C allele in *SELE* and rs6046AA genotype in *F7* (5.1 mmHg and 3.8 mmHg increase in SBP and DBP respectively) (Table 3A). We concluded that rs6046A might invert the BP-lowering effect of rs5355T on DBP and SBP.

**Table 3:** Blood pressure variations according to rs5355T allele in *SELE* and rs6046G/A genotypes in *F7* when compared to rs5355C allele in *SELE*.

A-Discovery population	*SELE*					
		rs5355T	P*		rs5355T	P*
		SBP (mmHg)			DBP (mmHg)	
***F7***	rs6046GG	−6.5		rs6046GG	−8	
	rs6046GA	6.1	0.047	rs6046GA	1.2	0.048
	rs6046AA	5.1		rs6046AA	3.8	
**B-Replication population**	***SELE***					
		**rs5355T**	P*		**rs5355T**	P*
		**SBP (mmHg)**			**DBP (mmHg)**	
***F7***	rs6046GG	−6.5		rs6046GG	–	
	rs6046GA	2.2	0.03	rs6046GA	–	0.102
	rs6046AA	3		rs6046AA	–	

Only significant blood pressure variations are shown.

BP variations in individuals carrying rs5355T allele in *SELE* and rs6046GG in *F7* were compared with carriers of rs5355C allele in *SELE* and rs6046GG genotype in *F7*. BP variations in individuals carrying rs5355T allele in *SELE* and rs6046GA genotype in *F7* were compared with carriers of rs5355C allele in *SELE*, rs6046GA genotype in *F7*. BP variations in carriers of rs5355T allele in *SELE* and rs6046AA genotype in *F7* were compared with those carrying rs5355C allele in *SELE* and rs6046AA genotype in *F7*.

DBP: diastolic blood pressure, P*: p value for epistatic interaction model, SBP: systolic blood pressure, BP: blood pressure.

In the replication population, rs6046G>A in *F7* was also associated with decreased SBP (P_replication_ = 8.45×10^−4^ and P_meta_ = 2.03×10^−4^) and DBP (P_replication_ = 2.58×10^−7^and P_meta_ = 9.16×10^−4^). In contrast, rs5355C>T was not associated with DBP (P_replication_ = 0.86). Most importantly, we found rs5355C>T in *SELE* and rs6046G>A in *F7* interacting in order to influence the SBP (P_replication_ = 0.03, Table 3B). Similar SBP variations according to rs5355T allele in *SELE* and rs6046G/A genotypes in *F7* were successfully found (Table 3B).

In conclusion, rs5355C>T in *SELE* interacted with rs6046G>A in *F7* in order to influence SBP in a total of 8,220 European individuals.

We investigated the eventual relation(s) between the epistatic interaction and the inflammation-related genes in a PBMCs model. rs5355C>T in *SELE* was not associated with any of the investigated transcripts. In contrast, rs6046A allele in *F7* was positively associated with *NAMPT* mRNA levels in both models (individual association and epistatic interaction models) (P = 9.2×10^−5^, β = 0.489 and P = 1.1×10^−5^, β = 0.552 respectively).

Increased *NAMPT* mRNA levels were positively correlated with *ICAM1* (P<1×10^−4^ and β = 0.576, Table 4), *SELL* (P = 5×10^−3^ and r = 0.308, Table 4), *FPR1* (P = 2×10^−4^ and r = 0.394, Table 4), *LL-37* (P = 4×10^−3^ and r = 0.452, Table 4) and *DEFA1-3* (P = 5×10^−3^ and r = 0.28, Table 4) genes expression. In addition *ICAM1*, *SELL*, *FPR1* and *DEFA1-3* expressions were also correlated (P≤5×10^−3^, Table 4). Only *DEFA1-3* and *LL-37* mRNA levels were positively associated with SBP. We found that both mRNAs explained 4% of SBP phenotypic variation (P = 3×10^−3^, β = 0.04 and P = 0.01, β = 0.03 respectively).

**Table 4:** Pearson’s correlations between *NAMPT, ICAM1, SELL, FPR1, DEFA1-3* and *LL-37* genes expression.

r P	*NAMPT*	*ICAM1*	*SELL*	*FPR1*	*DEFA1-3*	*LL-37*
***NAMPT***		0.6	0.3	0.4	0.3	0.5
						
***ICAM1***	<1×10^−4^		0.5	0.6	0.3	0.4
						
***SELL***	5×10^−3^	<1×10^−4^		0.697	–	–
						
***FPR1***	2×10^−4^	<1×10^−4^	<1×10^−4^		–	0.3
						
***DEFA1-3***	5×10^−3^	5×10^−3^	–	–		0.9
						
***LL-37***	4×10^−3^	1×10^−3^	–	<1×10^−4^	<1×10^−4^	

Only Significant correlations are shown (P≤5×10^−3^).

All genes expression were normalized to *POL2RA* mRNA levels.

r: Pearson’s correlation coefficient, P: P-value.

## Discussion

In the current study, we found rs6046A allele in *F7* associated with decreased BP levels (P≤3.7×10^−3^ and P_meta_≤2.03×10^−4^). In the discovery cohort, rs5355T allele in *SELE* was also associated with decreased DBP (P = 5×10^−3^).

rs6046G>A in *F7* was shown to be associated with increased F7 plasmatic levels [15]. More interestingly, this SNP was reported to have a role in protection against myocardial infarction in two different studies performed on Italian populations [29], [30]. rs5355C>T in *SELE* is located in chr.1q, a genomic region linked to BP related phenotypes in two independent linkage studies [31], [32]. These findings were supported by observation of mouse and rat BP-related quantitative trait loci in regions homologous to the human 1q chromosomal locus [33].

Herein, we showed that in a total of 8,220 European individuals, rs5355C>T in *SELE* interacted with rs6046G>A in *F7* and the latter SNP in order to alter SBP (P_discovery_ = 0.047 and P_replication_ = 0.03 respectively, Table 3). The above interaction was differently associated with SBP variations according to rs6046G>A genotypes (Table 3). In fact, epistatic interactions are phenomena where the effect of a gene is modified by another one [34], [35], thus although rs6046A allele in *F7* was associated with decreased BP levels, it interacted with rs5355T allele in *SELE* in order to influence SBP levels, resulting an increase in SBP mean values.

The non-replication of the association between rs5355C>T in *SELE* and DBP is not surprising as insignificant interaction effect on DBP between these two variants was found in the replication cohort. It is important to point out that, it has been postulated that epistatic interactions may identify genetic markers that are not captured by individual marker analysis and/or revealed by the combinatory effect of loci in other pathways [34], [35]. This postulate might explain why the two variants investigated here (and many others) were not reported among the top GWAS SNPs.

It was proven that blood coagulation factors enhance the inflammatory response leading to endothelial dysfunction accounting in part, for the vascular complications occurring in CVDs and their risk factors [36]. Thus, we searched for eventual relation(s) between the epistatic interaction and the inflammation-related genes in a PBMCs model. Numerous studies have revealed the importance of studying PBMCs in a strategy targeting the metabolic pathways of cardiovascular risk factors, such as HTN [5], [37], [38]. It has been recently demonstrated that PBMCs mRNA expression closely mimic the *in vivo* state and generate more physiologically relevant data concerning many health related traits [39]. The role of multiple metabolic pathways in HTN makes the study of PBMCs transcriptome important for the possible developing of diagnostic and prognostic tests [40], we assessed associations between rs5355C>T in *SELE* and rs6046G>A in *F7* with the inflammation-related genes expression. rs6046A allele in *F7* was associated with increased *NAMPT* mRNA levels (P≤9.2×10^−5^). *NAMPT* levels were also positively correlated with *ICAM1*, *SELL*, *FPR1*, *DEFA1-3* and *LL-37* genes expression (P≤5×10^−3^, Table 4). In addition *ICAM1*, *SELL*, *FPR1* and *DEFA1-3* expressions were also correlated (P≤5×10^−3^, Table 4). Only *DEFA1-3* and *LL-37* expressions were associated with SBP (P = 3×10^−3^ and P = 0.01 respectively) and explained 4% of its variation. Therefore, we suggest that the associations of *DEFA1-3* and *LL-37* mRNAs and SBP reflect the epistatic interaction and not the main effect of rs6046G>A in *F7*. Visfatin is a multifunctional protein that has been reported to be involved in innate immune system [41] and several other biological processes such as the cardiovascular system [42]. However, its role in BP was unclear. Supporting our results, three different *in vitro* studies have demonstrated that visfatin induced an endothelial dysfunction by increasing inflammatory and adhesion molecules expression such as ICAM1 [43]–[45]. In addition, in a previous study we have reported that gene expression of an antimicrobial peptide LL-37 in PBMCs was associated with altered BP levels [46]. The above findings support our epistatic and the *in vivo* results revealing an indirect link between *NAMPT* gene expression and BP through the expression of adhesion and innate immune system molecules.

**Figure 1:**
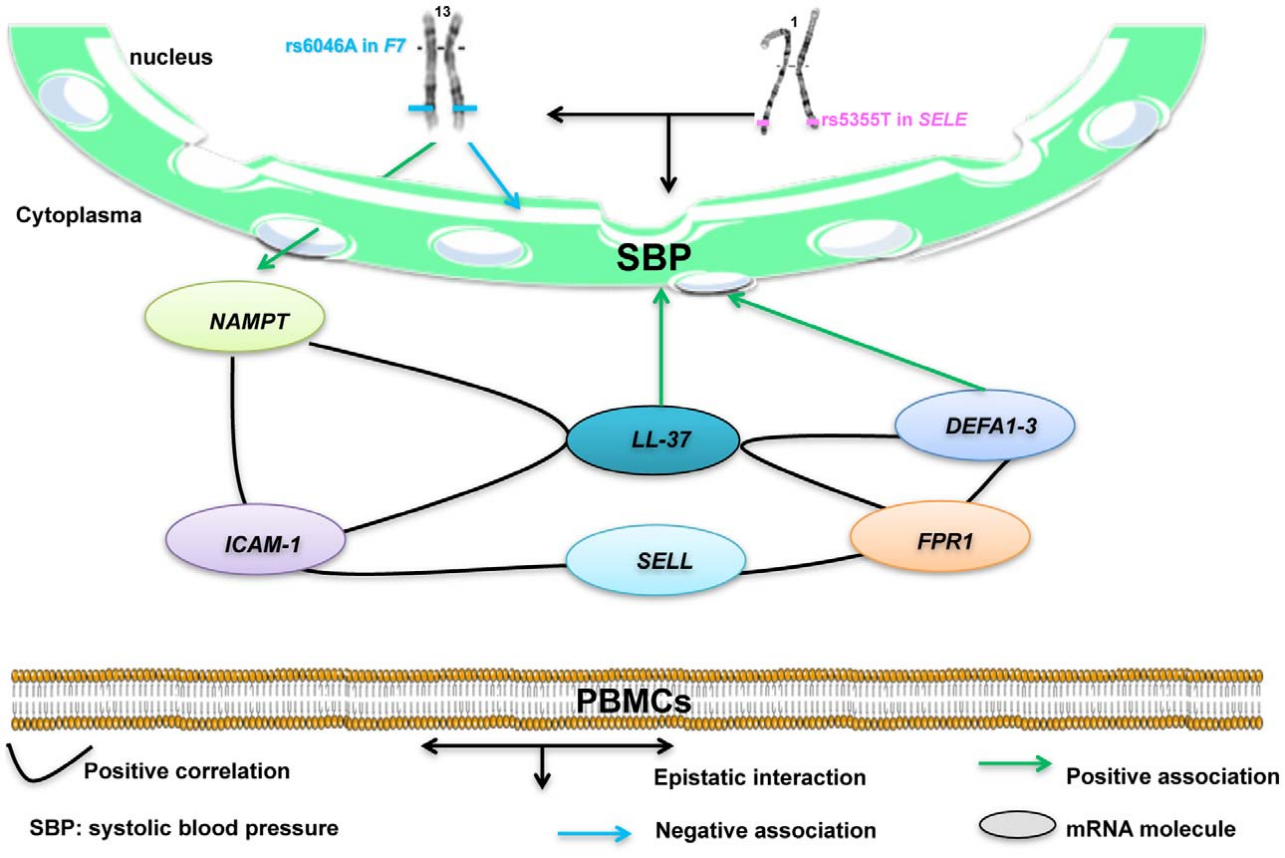
Summary of the study and hypothesis for rs5355C>T in *SELE* and rs6046G>A in *F7* interaction. rs6046A allele in *F7* was associated with decreased BP levels. rs5355C>T in *SELE* and rs6046G>A in *F7* interacted in order to alter SBP levels, rs6046A inverted the BP-lowering effect of rs5355T. rs6046A allele in *F7* was positively associated with increased *NAMPT* gene expression. *NAMPT* levels were positively correlated with *ICAM1*, *SELL*, *FPR1* and *DEFA1-3* genes expression. Only *DEFA1-3* and *LL-37* expressions were correlated and associated with SBP levels and explained 4% of its variation.

### Strengths and Limitations

The genetics of BP has never been easy [47]. For many years, it has been dominated by the stark contrast between its high heritability and the frustrating reality that no clearly reproducible and functional genetic variant could be discovered [3], with epistatic interactions accepted as cause of discrepancies across the studies.

The current study shows the first replicated epistatic interaction in the BP genetics field. This interaction between a coagulation factor gene (*F7*) and an adhesion molecule gene (*SELE*) is putatively functional through its link with five inflammation-related gene expression. Going in the same direction; it has been demonstrated that some blood coagulation factors can induce an endothelial dysfunction (*SELE* is a marker of endothelial dysfunction) through an inflammatory response accounting for the vascular complications occurring in CVDs and their risk factors [36]. Similarly, *NAMPT* expression was shown to increase the expression of inflammatory and adhesion molecules such as *ICAM1* [43]–[45]. Based on these findings we speculate a biological plausibility for the reported epistatic interaction. Supporting this statement, Tomaszewski et al [48] showed that a genetic risk score including SNPs from the fibroblast growth factor signaling pathway was able to explain a larger proportion of variation in HTN as compared with a genetic risk score including a similar number of SNPs based on the previous top SNPs from the GWAS [48]. This suggests that biological knowledge might support the reported epistatic interactions.

However, our study also had several limitations. Whereas focusing on European populations, our findings cannot be generalized to other ethnic groups. We also were unable to further investigate SNPs associations with plasmatic levels of the inflammation-related genes as the availability of biological materials was unfortunately limiting. Similarly, further studies looking at the SNPs association with *SELE* and *F7* expression in endothelial cells, would be of great value.

### Conclusion

Our findings are summarized in Figure 1. In European populations, we confirmed that rs6046A in *F7* is associated with decreased BP. Furthermore, we found that rs5355C>T in *SELE* and rs6046G>A in *F7* interacted in order to alter SBP levels. In addition rs6046A allele in *F7* was positively associated with increased *NAMPT* gene expression, which was linked with BP through inflammatory mechanisms via the expression of adhesion and innate immune system molecules.

### Perspectives

Even if additional investigations are needed, the present study highlighted the importance of taking into account candidate genes, GWAS and epistatic interactions in order to in deep investigate BP genetic regulation. One must also consider the functionality of relationships and G*E interactions that might be at the origin of the low until now predictive values of results in HTN. This integrative approach could better explain the missing heritability of this complex trait.

## Supporting Information

Supplementary Data S1 (DOC)
