# Supplementary material for: Functional Epistatic Interaction between rs6046G>A in F7 and rs5355C>T in SELE Modifies Systolic Blood Pressure Levels
Source: PLoS One. 2012 Jul 18;7(7):e40777. doi: 10.1371/journal.pone.0040777 (PMC3399862; doi:10.1371/journal.pone.0040777)
Supplement: Supplementary Data S1 — (DOC) [file pone.0040777.s001.doc]

**Functional epistatic interaction between rs5355C>T in *SELE and* rs6046G>A in *F7* modifies systolic blood pressure levels**

**Said El Shamieh^a^, MSc, Ndeye Coumba Ndiaye^a^, Ph.D, Maria G Stathopoulou^a^, Ph.D, Helena A Murray^b^, Ph.D, Christine Masson^a^, Bsc, John V Lamont^b^, Ph.D, Peter Fitzgerald^b^, Ph.D, Athanase Benetos^cd^, MD, Ph.D, Sophie Visvikis-Siest*^ad^, Ph.D.**

^a^ Université de Lorraine, “Génétique Cardio-vasculaire”, EA-4373, Nancy, F-54000, France.

^b^ Randox Laboratories Ltd, Crumlin, Antrim, United Kingdom.

^c^ INSERM U961, Université de Lorraine, Nancy, F-54000, France.

^d^ CHU Nancy, Brabois, Service de Gériatrie, Nancy, F-54000, France.

***Corresponding author:** Dr. VISVIKIS-SIEST Sophie, “Génétique Cardiovasculaire”, EA-4373, Université de Lorraine, Nancy, F-54000, France.

Tel: +33(0)6.07.60.25.69; fax: +33(0)3.83.32.13.22.

E-mail: [Sophie.Visvikis-Siest@inserm.fr](mailto:Sophie.Visvikis-Siest@inserm.fr)

**Table S1:** Summary of investigated genetic variants.

| **Locus** | **Gene** | **Chromosome** | **SNP ID** | **Position** | **Mutation** |
| --- | --- | --- | --- | --- | --- |
| *ACE* | Angiotensin I converting enzyme  (peptidyl-dipeptidase A) 1 | 17q23.3 | rs1799752 | 17:58919122-9629 | Ins>del |
| *CETP* | Cholesteryl Ester Transfer Protein | 16q21 | rs5882 | 16:55573293 | Val422Ile** |
| *SELE* | Selectin E | 1q22-25 | rs5355 | 1:167962494 | Leu575Phe* |
| *FGB* | Fibrinogen Beta chain | 4q28 | rs1800790 | 4:155703058 | 4577C>T |
| *FVII* | Coagulation factor VII | 13q34 | rs6046 | 13:113773159 | Arg353Gln |
| *LPL* | LipoProteinLipase | 8p22 | rs328 | 8:19863504 | Ser474X |
| *MMP3* | Matrix MetalloPeptidase 3 | 11q22.3 | rs3025058 | 11:102221062-1263 | T>Ins |
| *MTHFR* | 5,10-MethyleneTetraHydroFolate  Reductase | 1p36.3 | rs1801133 | 1:11778865-9065 | 825 A>G |
| *PON1* | Paraoxonase 1 | 7q21.3 | rs662 | 8:19863504 | Gln192Arg* |
| *TNF* | Tumor Necrosis Factor | 6p21.3 | rs1800629 | 6:31650510 | 4682 G>A |

The prediction of possible nsSNPs impact on the structure and function of its specific protein was performed using PolyPhen.

*: benign nsSNP.

**: damaging nsSNP.

**Table S2:** Primer sequences and qPCR conditions.

| **Target transcripts** | **Forward primer (5’-3’)** | **Reverse primer (5’-3’)** | **Annealing (^o^C, sec)** | **PCR product size**  **(pb)** |
| --- | --- | --- | --- | --- |
| *LL-37* | CTCGGATGCTAACCTCT | CATACACCGCTTCACC | 61^o^C, 10sec | 178 |
| *DEFA1-3* | GCGGACATCCCAGAAGTGGTTG | TCAGCAGCAGAATGCCCAGAGTC | 58^o^C, 10sec | 174 |
| *FPR1* | TGGACCAACGACCCTAA | AAGGCTGCTGCGACAA | 59^o^C, 10sec | 203 |
| *ICAM1* | CCCCGGTATGAGATTG | ATGCGTGGCCTAGTGT | 54^o^C, 5sec | 370 |
| *SELE* | GCCTGCTACCTACCTGTGAA | GGCAGGAACAAATTTCTTTGC | 60^o^C, 30sec | 149 |
| *SELL* | GGAACATCTTCAAGTTGTGG | GTGTAATTGTCTCGGCAG | 62^o^C, 10sec | 142 |
| *SELP* | CTTAATGGCTCTGCACAAAC | CGCTCCACCAAAGTAAGTC | 62^o^C, 10sec | 117 |
| *NAMPT* | TGTGGTCAGCGATAGC | AAGATAAGGTGGCAGC | 72^o^C, 8sec | 220 |
| *LEP* | GTGCGGATTCTTGTGG | GACTTTCTGTTTGGAGGA | 72^o^C, 6sec | 154 |
| *TNF* | TCTACTTTGGGATCATTGCC | GTGGTGGTCTTGTTGCTTAA | 72^o^C, 7sec | 182 |
| *IL-6* | TACCCCCAGGAGAAGATTCC | GCCATCTTTGGAAGGTTCAG | 60^o^C, 5sec | 199 |
| *POLR2A* | CAGACCGGCTATAAGGTGGA | GGTAGACCATGGGAGAATGC | 57^o^C, 5sec | 123 |

**Table S3:** Clinical characteristics of the subsample used for gene expression analysis in PBMCs.

|  | TOTAL |
| --- | --- |
| N (% women) | 90 (49.9) |
| Age (years) | 50.9 ± 4.5 |
| BMI (kg/m²) | 25.6 ± 3.8 |
| SBP (mmHg) | 120.2 ± 14.8 |
| DBP (mmHg) | 71.6±9.3 |

BMI: body mass index, SBP: systolic blood pressure, DBP: diastolic blood pressure.

**Table S4:** Individual associations results with blood pressure.

|  | | | **Discovery population** | | | | | **Replication population** | | | | |
| --- | --- | --- | --- | --- | --- | --- | --- | --- | --- | --- | --- | --- |
|  |  |  | **SBP** | | **DBP** | | **HWE** | **SBP** | | **DBP** | | **HWE** |
| **CHR** | **SNP** | **GENE** | **P** | **Beta** | **P** | **Beta** | χ²***** | **P** | **Beta** | **P** | **Beta** | χ² |
|  |  |  |  | **(mmHg)** |  | **(mmHg)** |  |  | **(mmHg)** |  | **(mmHg)** |  |
| 17 | rs1799752 | *ACE* | 0.92 | - | 0.775 | - | 0.46 | - | - | - | - | - |
| 1 | rs5355 | *SELE* | 0.021 | - | 0.005 | -0.04 | 2.03 | - | - | 0.86 | - | 0.05 |
| 16 | rs5882 | *CETP* | 0.094 | - | 0.078 | - | 3.05 | - | - | - | - | - |
| 4 | rs1800790 | *FGB* | 0.304 | - | 0.438 | - | 0.2 | - | - | - | - | - |
| 13 | rs6046 | *F7* | 0.004 | -0.06 | 0.001 | -0.08 | 0.02 | 8.45x10^-4^ | -0.06 | 2.58x10^-7^ | -0.08 | 0.5 |
| 12 | rs5443 | *GNB3* | 0.611 | - | 0.474 | - | 0.49 | - | - | - | - | - |
| 8 | rs328 | *LPL* | 0.051 | - | 0.085 | - | 3.23 | - | - | - | - | - |
| 11 | rs3025058 | *MMP3* | 0.126 | - | 0.064 | - | 0.01 | - | - | - | - | - |
| 1 | rs1801133 | *MTHFR* | 0.932 | - | 0.937 | - | 3.37 | - | - | - | - | - |
| 7 | rs662 | *PON1* | 0.352 | - | 0.434 | - | 2.87 | - | - | - | - | - |
| 6 | rs1800629 | *TNF* | 0.499 | - | 0.467 | - | 3.17 | - | - | - | - | - |

Beta coefficients are shown for significant associations.

CHR: chromosome, SNP: single nucleotide polymorphism, Beta: coefficient in the linear regression model, BP: blood pressure, SBP: systolic blood pressure, DBP: diastolic blood pressure.

*χ² test for Hardy-Weinberg deviation at one degree of freedom (dof). Signification threshold at 5% is 3.84.
